# Evidence for a role of hemozoin in metabolism and gametocytogenesis

**Published:** 2017-08-01

**Authors:** Ghazi A. Jamjoom

**Affiliations:** 1 College of Applied Medical Sciences, and King Fahd Medical Research Center, King Abdulaziz University, Jeddah, P.O. Box 415 Jeddah 21411, Saudi Arabia

## Abstract

Hemozoin is generally considered a waste deposit that is formed for the sole purpose of detoxification of free heme that results from the digestion of hemoglobin by *Plasmodium* parasites. However, several observations of parasite multiplication, both in vertebrate and invertebrate hosts are suggestive of a wider, but overlooked, metabolic role for this product. The presence of clinical peripheral blood samples of *P. falciparum* with high parasitemia containing only hemozoin-deficient (non-pigmented) asexual forms has been repeatedly confirmed. Such samples stand in contrast with other samples that contain mostly pigmented circulating trophozoites and gametocytes, indicating that pigment accumulation is a prominent feature of gametocytogenesis. The restricted size, i.e. below detection by light microscopy, of hemozoin in asexual merozoites and ringforms of *P. falciparum* implies its continuous turnover, supporting a role in metabolism. The prominent interaction of hemozoin with several antimalarial drugs, the involvement of proteins in hemozoin formation, and the finding of plasmodial genes coding for a heme-oxygenase-like protein argue for a wider and more active role for hemozoin in the parasite’s metabolism. The observed association of hemozoin with crystalloids during ookinete development is consistent with a useful function to it during parasite multiplication in the invertebrate host. Finally, alternative mechanisms, other than hemozoin formation, provide substitute or additional routes for heme detoxification.

## 1 Introduction

Hemozoin (malaria pigment) is a crystalline dimer of β-hematin (ferriprotoporphyrin IX) that is formed upon digestion of hemoglobin by the malaria parasite [1-6]. The long-held view is that the formation of hemozoin serves in the detoxification of free heme molecules by converting them into an inert crystalline form. However, several observations have accumulated in the past years that indicate that the role of hemozoin is not likely to be restricted to this aspect. Some of these observations have led me previously to an alternative hypothesis in which hemozoin, which prominently accumulates during sexual differentiation, is likely to serve a useful function for the parasite in the invertebrate host [7,8]. Hemozoin's deficiency in various samples of *P. falciparum* indicate that it is continuously formed and broken down in the metabolically active asexual multiplication stages [8]. This hypothesis is re-visited here in light of recent independent supportive data.

## 2 Hemozoin-deficient and hemozoin-rich peripheral blood samples of *P. falciparum*

### 2.1 Pigment-deficient samples

The presence of frequent clinical samples of *P. falciparum* in which no hemozoin can be detected by conventional Giemsa staining or more sensitive techniques such as dark-field microscopy, as reported previously [7], has now been confirmed in recent studies. Thus, Delahunt *et al.* [9] described samples from *P. falciparum*-infected patients in whom only hemozoin-deficient young ring-stage parasites are detectable, even at very high levels of parasitemia that may exceed 10%, reflecting the occurrence of multiple cycles of replication. Such samples constitute a significant percentage of the total number of clinical samples examined (5/10 in Delahunt’s study [9] and 25/45 in my previous work [7]). Yet no hemozoin pigment is detectable in the parasite forms circulating in blood, which are mostly ring-forms and young trophozoites. In these two studies the technique of dark-field microscopy (DF), which is considered the gold standard for hemozoin detection was used. While my study relied on manual examination, Delahunt *et al.* [9] used image analysis software that recognises hemozoin, for cell-by-cell comparison between DF-examination and conventional Giemsa-staining.

Using a different technique, i.e. flow cytometry, Rebelo *et al.* [10], reported the absence of detectable hemozoin in ten blood samples from patients with *P. falciparum* in whom parasitemia (as determined by SYBR green and Giemsa staining) ranged from 0.5 - to 7%.

These studies confirm the century-old observation of *P. falciparum* samples lacking pigmented forms (Bignami and Bastianelli – cited by Sherman *et al.* [11]. Still, the inability to detect pigment by the more sensitive techniques such as DF microscopy and flow-cytometry came as a surprise.

Despite their rarity in peripheral blood due to such factors as sequestration (see below), rupture, or phagocytosis [12], asexual pigmented forms of *P. falciparum*, e.g. schizonts or pre-schizont trophozoites, are still occasionally observable, especially in very heavy infections. It should therefore be emphasised that the failure to detect such forms in pigment-deficient samples may not completely rule out their presence at a level that is below the sensitivity of the techniques used.

Finally, the inability to detect hemozoin in pigment-deficient samples of *P. falciparum* does not indicate that it is not formed at all but only that it does not accumulate to the size required for its detection by the aforementioned techniques used. This point can be resolved by electron microscopy by which hemozoin formation is generally observable in similar samples in close association with hemoglobin digestion in the food vacuole [13,14]. In addition, hemozoin accumulation in pigment-deficient samples can be induced by several factors, e.g. incubation in tissue culture medium or treatment with various agents such as Tween 20 or Jasplankinolide (see below).

### 2.2 Pigment-rich samples

The pigment-deficient samples described above stand in stark contrast with other clinical peripheral blood samples of *P. falciparum* in which the majority of circulating forms are more developed ring-forms of trophozoites containing easily detectable pigment [7]. These samples became more noticeable with techniques directed at hemozoin pigment detection, such as DF microscopy although their occurrence in field specimens is not rare (38% in my study [7], and 3/10 in Delahunt *et al.* [9]). They are also recognisable in Giemsa stained blood films as samples containing more developed ring-form trophozoites (CDC DPx slides [15]). Pigmentation in this type of samples is considered to reflect the normal expected pattern of maturation. Therefore, these samples did not raise any curiosity as in the case of pigment-deficient samples. However, the occurrence of these two clearly-distinguishable patterns of pigmentation (hemozoin-deficient and hemozoin-rich) of the same species of parasite calls for a satisfactory explanation. Less frequently, a mixed pattern of hemozoin-containing and hemozoin-lacking trophozoites is observed [7,9].

## 3 Sequestration

Sequestration, i.e. the sticking of erythrocytes infected with *P. falciparum* parasites to the endothelium of the inner capillaries has long been recognised [11] and is an essential feature of severe *P. falciparum* pathology [16,17]. It is considered a main factor for the rare appearance of schizonts and pre-schizont mature (pigmented) trophozoites in peripheral blood during the asexual development cycle of this species.

Schizonts contain a significant amount of clumped pigment and are thus easily detectable by sensitive pigment-detection techniques such as DF. Their relative rarity in peripheral blood may thus be adequately explained by sequestration. The situation differs for mature (pigment-containing) trophozoites. These forms exhibit two distinct behaviours in *P. falciparum*, i.e. complete absence from peripheral blood in pigment-deficient samples and frequent presence in pigment-rich samples. These behaviours can best be explained by proposing two types of mature trophozoites, i.e. those that will become schizonts and those that will differentiate into gametocytes. Sequestration can explain the rarity of the first type in pigment-deficient samples. On the other hand, the abundantly present pigmented trophozoites in pigment-rich samples will be difficult to account for as a stage of the asexual replication cycle and must be considered as a different, second type. This dilemma can only be resolved by proposing that these second type trophozoites represent stages of sexual differentiation.

In conclusion, sequestration cannot explain the complete absence of pigmented trophozoites in some samples of *P. falciparum* and their abundance in other samples. For sequestration to explain this observation, it has to be almost 100% efficient, in the first type of samples and to completely cease working in the second type.

## 4 Pre-schizont and pre-gametocyte trophozoites

In order to understand the above-described two common patterns of pigmentation in *P. falciparum* samples, distinction must be made between mature trophozoites committed to either of the asexual or sexual pathways. Mature trophozoites are defined here as those that contain easily-visible hemozoin pigment. Table 1 summarises the expected properties of these two types of mature pigmented trophozoites. The distinction between pre-schizont and pre-gametocyte trophozoites will be reflected in their external antigens as revealed by stage-specific antibodies and by surface receptors that result in their binding to endothelium [18].

**Table 1:** Pigmented trophozoites in *P. falciparum.*

Type	Pre-schizont	Pre-gametocyte
Multiplication	Asexual	Sexual
Destination	Schizont	Gametocyte
Frequency in peripheral blood	Rare	Common
Sequestration	Yes	No
Pigment location	Residual body	Body of trophozoites
Pigment clumping	Yes	No

## 5 Synchronisation

Synchronisation in *P. falciparum* generally refers to the shift in the pattern of parasite in peripheral blood from non-pigmented rings or young trophozoites to mature pigmented trophozoites. There are two possibilities for the, apparently synchronised, transition between a pigment-deficient pattern and a pigment-rich pattern. One possibility is that this transition occurs during asexual multiplication, the other is that it is a feature of sexual development.

In the first possibility, pigment-deficient and pigment-rich types of *P. falciparum* may represent synchronised consecutive broods of asexual maturation stages on the way to schizogeny. Synchronised asexual multiplication can be induced in cell culture with sorbitol treatment in which pigment becomes visible after 30 hours [17] or earlier (10-16 hrs) if viewed by DF microscopy [9].

However, in clinical samples containing high levels of parasitemia, many random cycles of multiplication must have occurred making it unrealistic to achieve such a tight level of synchronisation so as to produce samples with almost complete pigment deficiency. No such tight synchronisation is observed with other species of human malaria. In fact, synchronisation is less expected in *P. falciparum* in which the rhythm of schizogeny is frequently less regular than in other species [19]. In heavy *P. falciparum* infection, all stages of asexual multiplication (ringforms, trophozoites, schizonts) are simultaneously observed [20]. Immune serum can reverse sequestration [21]. These observations do not support tight synchronisation as a cause of pigment-rich samples being a stage of asexual development.

In the likely absence of complete synchronisation of asexual parasite division, the mixed presence of both pigment-deficient and pigment-containing trophozoites would be expected in all clinical samples if pigment-rich trophozoites were part of asexual development. As this is not the case, a different mechanism, other than synchronisation, must therefore be operative to account for the observed occurrence of totally pigment-deficient samples.

**Figure 1:**
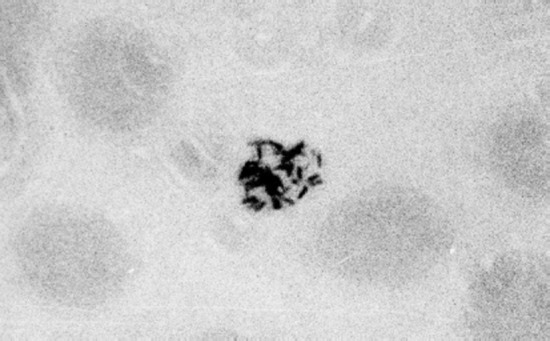
Pigment rodlets in an unstained preparation of *P. falciparum* gametocyte (x1000).

In distinction to asexual multiplication, two observations support the idea that synchronised, pigment accumulation in the trophozoites of pigment-rich *P. falciparum* samples constitutes a stage along sexual differentiation. First, the mature sexual forms, gametocytes, contain a large amount of distinct pigment rodlets, their number reaching 30-40, which accumulate in the cytoplasm during gametocyte development (Figure 1). This is also the case in trophozoites of pigment-rich samples in which pigment accumulates in the body of the trophozoites. In schizonts, in contrast, pigment accumulates only in the residual body (see below).

Second, gametocytes and pigment-rich trophozoites both persist in peripheral blood, which is a necessary condition for gametocyte transmission to the mosquito vector. This is in distinction with schizonts, and, presumably, pre-schizont trophozoites, which tend to sequester within internal tissues. Finally, the fate of pigmented trophozoites (either as pre-schizont or pre-gametocyte) could be determined by staining with stage-specific monoclonal antibodies [18].

## 6 Pigment location in *P. falciparum* schizonts

In schizonts, pigment appears outside the boundaries of the forming daughter merozoites, i.e. in the region designated ‘residual body’ [14]. Pigment increases and clumps within the vacuole of the residual body. The feeding apparatus (cytosome) of the original mother cell remains associated with the residual body while daughter merozoites are separated by their individual cell membranes. The residual body is devoid of a nucleus but maintains active hemoglobin degradation. A significant amount of hemozoin accumulates in the residual body. Consistent with the widely held view, this pigment behaves as an inert waste product that is released by cell rupture. Pigment in daughter merozoites or in the subsequent early ringforms or early asexual trophozoites of *P. falciparum* does not accumulate to a detectable level by BF or DF microscopy. This indicates the presence of a fundamental difference in the mechanism of pigment accumulation between the asexual and sexual cycles.

The presence of pigment in the schizont, as such, indicates that the mechanism of hemozoin synthesis is functioning in the asexual cycle as it is in the sexual cycle leading to gametocytogenesis. However, the accumulation of pigment only in the residual body of the schizont, but not in the metabolically-active dividing merozoites or subsequent rings and early trophozoites suggests that hemozoin is most likely formed but broken down in merozoites and early rings before it reaches a size detectable by light microscopy (see below). Less likely, pigment accumulation in the merozoites and rings of *P. falciparum*, in contrast to schizonts, may cease before hemozoin reaches a detectable level, without necessarily being utilised. Besides, no system of pigment secretion or transfer from merozoites to the residual body has been observed, in contrast to hemoglobin transfer in malaria where an extensive actin-dependent system has been described [22].

## 7 Commitment to gametocytogenesis

The large amount of pigment seen in the gametocyte indicates that pigment accumulation must proceed in the trophozoites that are committed to gametocytogenesis. Sexual commitment to gametocytogenesis is a major development in the life-cycle of the malaria parasite. A collective shift from an asexual multiplication to gametocytogenesis, i.e. waves of gametocytemia, in a multiplying parasite population have been observed and attributed to host immune response, or haematological factors [23-26]. Diffusible factors, e.g. phorbol diesters, cAMP, antimalarial drugs, etc. have been suggested to induce gametocytogenesis by activating specific signal transduction pathways [26].

Commitment to gametocytogenesis is also affected by the parasite's own genetic programme. Recently, Kafsack *et al.* [27] showed that the expression of the DNA binding protein PfAP2-G correlates strongly with gametocyte formation. This protein is activated by a transcriptional switch to serve as a master regulator of sexual development.

A collective shift to gametocytogenesis offers the most plausible explanation for the two patterns of pigmentation of *P. falciparum*, i.e. pigment-deficient and pigment-rich. Circulating pigmented trophozoites in pigment-rich samples would thus represent the expected precursors to gametocytes [7,8]. The occurrence of these forms in bulk is consistent with induction by a diffusible environmental host factor.

## 8 Asexual multiplication and sexual differentiation

There are two recognised types of intraerythrocytic multiplication of the malaria parasite, the asexual cycle leading to vegetative multiplication and the sexual maturation for the formation of gametocytes.

**Figure 2:**
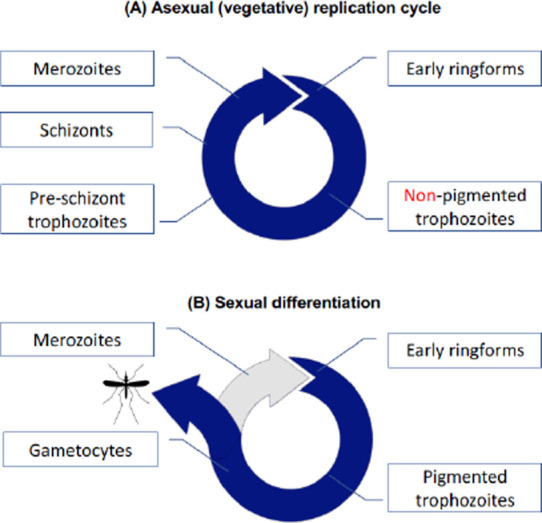
Asexual multiplication cycle (A) and sexual differentiation (B) in *P. falciparum*.

Currently, both the pigment-deficient and pigment-containing trophozoite forms are presumed to be part of both asexual and sexual cycles. However, according to the above-mentioned observation of two different patterns of pigmentation in *P. falciparum*, the currently accepted step from circulating pigmented trophozoite to schizont must be cancelled (Figure 2).

In other species of malaria it is assumed that pigment is seen in ringforms or asexual trophozoites because it reaches a larger size. However, in all species, it cannot be ascertained that any observed pigmented trophozoite is not destined for sexual differentiation unless special techniques are used to distinguish between asexual and sexual trophozoites.

## 9 Pigment utilization

The fact that hemozoin is limited to a small size in growing asexual stages of *P. falciparum* but enlarges in the residual body of schizont and during sexual differentiation is best explained by its continuous formation and breakdown during asexual multiplication. Evidence for the breakdown of hemozoin in these forms is a testable hypothesis. A breakdown hypothesis may invoke re-examination of the relationship of hemozoin to protein or enzymatic factors in the parasite. Such factors may be essential for a controlled mechanism of breakdown. While the currently accepted structure of hemozoin favours a non-enzymatic process for its crystallization, the discovery of a protein that accelerates the process of hemozoin formation (re-labelled Heme Detoxification Protein-HDP [28]) has lent support to a role for an enzyme-driven process. Involvement of enzymes in accelerating hemozoin formation would make it easier to envisage a mechanism for reversing this process, thereby leading to controllable degradation.

## 10 Hemozoin in the invertebrate host

Hemozoin remains visible in malarial stages in the mosquito gut [8]. It has generally been observed in association with crystalloid particles in the ookinete and oocysts [29]. Such an association is interesting and needs to be further investigated. Crystalloids have recently been suggested to play an important role in protein trafficking and sporozoite development [29].

## 11 Hemozoin and antimalarial drugs

Several of the main antimalarial drugs, including chloroquine and artemisinin, act via inhibition of hemozoin production [1,30-40]. Other agents with antimalarial activity (e.g. clometrazole [41], quaternary ammonium compounds [42]) similarly act by inhibiting hemozoin formation. The inhibition of β-hematin formation has been taken as a mechanism for killing the parasite by preventing the detoxification of free heme. It remains possible that any other metabolic role for hemozoin may also be affected by the actions of these drugs and agents.

## 12 Alternative mechanisms for heme detoxifycation

The generally acknowledged role in heme detoxification is not exclusive to hemozoin. Other mechanisms include heme degradation by glutathione [41,43-45], its neutralisation by binding to histidine-rich protein 2 [46], and, as discussed below, degradation by heme-oxygenase.

## 13 Possible role of hemozoin

If hemozoin is dynamically formed and broken down during vegetative multiplication, this suggests that it may serve as a useful metabolite for the parasite. Hemozoin's accumulation during the sexual differentiation phase suggests that it may also serve a useful role in the mosquito, perhaps as a source of some essential products. Before the latest structural studies [1-3], hemozoin was assumed to be a hemeoprotein [1,2]. Therefore, it could be proposed that it provided the parasite with part of its amino acid requirement in the mosquito [8]. However, in light of the current view of hemozoin as a crystal of β-hematin, focus may be narrowed to the heme molecule with its components of the tetrapyrrol backbone and iron molecule, although the described binding of histidine rich protein to heme and its proposed mediation of hemozoin formation [47] brings back the possibility of an associated protein component.

Heme is an essential co-factor that is required for diverse metabolic processes. Despite the plentiful supply of heme from host hemoglobin degradation, the malaria parasite is able to synthesise its own heme [48]. Heme oxygenase (HO) enzymes are broadly expressed by many organisms to degrade heme for disposal, to process it for metabolic utilisation of the tetrapyrrole backbone, or to release and scavenge the protoporphyrin-bound iron [49]. Okada [50] identified a heme oxygenase-like (HO) sequence in the genome of *P. falciparum*. The coding by the parasite of such an activity may be relevant to the proposal of utilisation of the heme content of hemozoin particularly in special circumstances, such as in the mosquito.

The high concentration of heme iron that results from extensive hemoglobin digestion by the parasite is considered to be a major threat to parasite survival through the possibility of generating oxygen free radicals. Detoxification of free heme iron by incorporating it into hemozoin crystals is currently considered to be the main mechanism for iron detoxification [51,52]. On the other hand, despite this high availability of heme iron, the parasite is killed by low concentrations of iron chelators indicating that the amount of bioavailable iron is limited and crucial for parasite growth [53,54]. The *Plasmodium* parasite requires iron for DNA synthesis, glycolysis, pyrimidine synthesis, heme synthesis and electron transport.

The availability of iron during the invertebrate phase of growth is not well known. It is not inconceivable that hemozoin stores may contribute to iron bioavailability in the mosquito stages of the parasite.

## 14 Conclusions

Pigment deficient samples of *P. falciparum* constitute an anomaly to the expected early accumulation of pigment based on synchronised culture and suggest the continuous utilisation of hemozoin, preventing its build-up to a detectable level in the circulating asexual stages.

Pigment-rich circulating trophozoites commonly seen in other samples of *P. falciparum* are unlikely to be intermediate stages in the asexual cycle but most likely represent pre-gametocytic stages. Pigment accumulation in circulating trophozoites is apparently triggered by the mechanism that initiates sexual differentiation leading to gametocytogenesis. Several observations are suggestive of a role for hemozoin in providing useful metabolites to the parasite especially in the invertebrate host, in distinction to its role in heme detoxification. Future studies using stage-specific antibody labels or radioactive tracing may directly test various aspects of this hypothesis.
